# Palliative care in primary care: European Forum for Primary Care position paper

**DOI:** 10.1017/S1463423619000641

**Published:** 2019-09-18

**Authors:** Danica Rotar Pavlič, Diederik Aarendonk, Johan Wens, José Augusto Rodrigues Simões, Marie Lynch, Scott Murray

**Affiliations:** 1 Assistant Professor, Department of Family Medicine, University of Ljubljana, Ljubljana, Slovenia; 2 Coordinator, European Forum for Primary Care, Utrecht, the Netherlands; 3 Professor of General Practice/Family Medicine, Senior University Lecturer, Research director, Department of Primary and Interdisciplinary Care, Faculty of Medicine and Health Sciences, University of Antwerp, Antwerpen, Belgium; 4 Professor, Department of Medical Sciences, University of Beira Interior, Covilhã, Portugal; 5 Programme Development Manager, The Irish Hospice Foundation, Dublin, Ireland; 6 St Columba’s Hospice Professor of Primary Palliative Care, University of Edinburgh and Co-Chair, European Association of Palliative Care Primary Care Reference Group, Emeritus Professor of Primary Palliative Care, University of Edinburgh,Edinburgh, Scotland

**Keywords:** palliative care, position paper, primary care

## Abstract

**Aim:**

The aim of this position paper is to assist primary health care (PHC) providers, policymakers, and researchers by discussing the current context in which palliative health care functions within PHC in Europe. The position paper gives examples for improvements to palliative care models from studies and international discussions at European Forum for Primary Care (EFPC) workshops and conferences.

**Background:**

Palliative care is a holistic approach that improves the quality of life of patients and their families facing problems associated with terminal illness, through the prevention and relief of suffering by means of early identification and diligent assessment and treatment of pain and other problems, whether physical, psychosocial, or spiritual. Unfortunately, some Europeans, unless they have cancer, still do not have access to generalist or specialist palliative care.

**Methods:**

A draft of this position paper was distributed electronically through the EFPC network in 2015, 2016, and 2017. Active collaboration with the representatives of the International Primary Palliative Care Network was established from the very beginning and more recently with the EAPC Primary Care Reference Group. Barriers, opportunities, and examples of good and bad practices were discussed at workshops focusing on palliative care at the international conferences of Southeastern European countries in Ljubljana (2015) and Budva (2017), at regular conferences in Amsterdam (2015) and Riga (2016), at the WONCA Europe conferences in Istanbul (2015), Copenhagen (2016), and Prague (2017), and at the EAPC conference in Madrid (2017).

**Findings:**

There is great diversity in the extent and type of palliative care provided in primary care by European countries. Primary care teams (PCTs) are well placed to encourage timely palliative care. We collected examples from different countries. We found numerous barriers influencing PCTs in preparing care plans with patients. We identified many facilitators to improve the organization of palliative care.

## Introduction

Although preserving life is a central goal of medicine, in the end, death is an unavoidable outcome. Exposure to death and dying has a major impact on one’s current quality of life (Sinclair, 2011). With the number of deaths predicted to rise dramatically in the future, the current high percentages of hospital deaths are difficult to sustain. An expansion of palliative care provision will need to take place in all settings and, importantly, at the primary health care (PHC) level, close to patients’ homes (Claessen, et al., 2013; Gomes, et al., 2013). Palliative care is a holistic approach that improves the quality of life of patients and their families facing problems associated with life-limiting illnesses. It offers the prevention and relief of suffering by means of timely identification and diligent assessment and treatment of pain and other problems, whether physical, psychosocial, or spiritual (World Health Organization, 2018). However, medical interventions are sometimes delivered in a fragmented pattern; that is, only individual components of palliative care (eg, pain medication, home parenteral nutrition, home oxygen, psychotherapy, social work, bereavement support, respite care, physical exercise, assistance with living wills, etc.) are available to some populations. This fragmentation also indicates the necessity for palliative care to be fully coordinated and integrated into health care delivery within every setting and to include PHC. Throughout Europe, palliative care is being developed in various ways, both as a specialty and also by generalist doctors and nurses. Unfortunately, some Europeans, unless they have cancer, still do not have access to generalist or specialist palliative care (Murray et al., 2015).

### How many people are dying at home?

National discussions on the place of death are taking place in all developed countries, in specialist services, in nursing homes and hospitals, and among the general public. There is increasing medicalization of dying, and the trend is for increasing numbers of deaths to occur in hospitals Sarmento, (2016). Despite efforts to develop policies that enable more people to die at home, only a minority of deaths do take place at home: only 17% in Norway in 2008, 21% in England in 2010, 23% in Belgium in 2007, 30% in Canada in 2004, 33% in Portugal in 2005, 34% in Italy in 2002, and 34% in the Netherlands in 2003 (Gomes and Higginson, 2006; Cohen et al., 2010; Houttekier et al., 2011; Gomes, 2012). Slovenia’s National Institute of Public Health statistics show that 51.2% of people died in hospitals in 2013. Czech and Slovak patients with chronic conditions are more likely to die in hospitals compared with those in some other European Union member countries (58.4% in the Czech Republic, 54.8% in Slovakia; Martin Loucka, et al., 2014). In the Netherlands, many cancer patients with end-stage disease prefer to die at home. In Ireland, although two-thirds of people express a wish to die at home, only approximately 25% of the population actually do so (Murray, 2013). Place of death is not just a matter of personal decisions, but it depends on what supports are in place to allow a person to die at home, such as presence of a caretaker, nature of the illness, symptoms associated with it, their socioeconomic and demographic status, and the availability of quality local services (Murray, 2013; Beernaert, et al., 2014; Loucka, et al., 2014).

### The role of PHC

PHC has a great potential to deliver effective palliative care to patients, especially home-based palliative care (Gomes, et al., 2013). Members of primary care teams (PCTs) are also responsible for the coordination and continuity of care, care decision-making, and referral to secondary care such as specialist palliative care services (Beernaert, et al., 2014). Most of the time, patients appreciate the contribution of PCTs (GPs, district nurses, physiotherapists, social workers, hospices, and lay support), especially if they are accessible, take time to listen, allow patients and carers to express their feelings, and make efforts regarding symptom relief. The importance of the relationship, based on continuity and a personal connection, remains a central focus for PCTs. Community-oriented PHC and the integration of various primary care disciplines are important for the quality of palliative care. However, reports from bereaved relatives suggest that palliative care is performed less well in the community than in other settings (Mitchell, 2002). PCTs that know the patients and their social context could play a key role in providing this early anticipatory palliative care (Claessen et al., 2013; Gomes, et al., 2013). The traditional focus on specialist palliative care teams caring for people in a hospice or community setting has been expanded (Radbruch and Payne, 2009; Radbruch and Payne, 2010; Watson et al., 2014).

### Aim of this position paper

The aim of this position paper is to assist PHC providers, policymakers, and researchers by discussing the current context in which health care for palliative patients functions within PHC in Europe. The position paper seeks to discuss changes in service models, policy, education, and research in the PHC context. A wider examination of home palliative care models and a critical appraisal of the variation in findings will help improve the evidence base for the development, implementation, and evaluation of home palliative care services in the future. It gives evidence for improvements to patient, family, and health systems available for different population-based models of palliative care from studies and international discussions at workshops and conferences.

## Methods

A draft of this position paper was distributed electronically with additional questions through the European Forum for Primary Care (EFPC) network in 2015, 2016, and 2017. The first goal was to identify how various models of palliative care had been defined in the literature and which of these were supported by evidence. Inclusion criteria relating to palliative care were based on the World Health Organization (WHO) definition, which is the most widely used internationally (World Health Organization, 2013). Active collaboration with representatives of the International Primary Palliative Care Network was established from the very beginning and more recently with the EAPC Primary Care Reference Group. Barriers, opportunities, and examples of good and bad practices were discussed at workshops focusing on palliative care in PHC. The EFPC held workshops at the international conferences of Southeastern European countries in Ljubljana (2015) and Budva (2017), at regular conferences in Amsterdam (2015) and Riga (2016), at the WONCA Europe conferences in Istanbul (2015), Copenhagen (2016), and Prague (2017), and at the EAPC conference in Madrid (2017). The topics discussed were the following:

- The role of PCTs;
- The needs assessment based on how many people are dying at their homes;
- Barriers and facilitators;
- Education in PHC;
- Research.

The position paper was then further refined to contain relevant resource material and was finally approved as a practical document by the executive board members of EFPC.

## Findings

There is a great diversity in the extent and type of palliative care in primary care provided by European countries. Examples from different countries were collected and are presented in boxes.

The German Association for Palliative Medicine received an award for its ‘Integrated hospice and palliative care in the district of Mühldorf am Inn: Time-intensive care’ project in 2016. The work received the award because it addresses challenges of palliative care in the community with a comprehensive concept of networking across care sectors and districts. The emphasis of this project is hence more on care delivery than on research.

Irish policy acknowledges the provision of palliative care by PCTs (DOHC 2001; IHF 2011). A community intervention team (CIT) includes nurses with enhanced clinical skills who provide a rapid and integrated response to a patient with an acute episode of illness for a defined period of time. This may be provided at home, in a residential setting, or in the community as deemed appropriate, thereby avoiding acute hospital admission. CIT personnel have a strong liaison role with hospital and community clinicians, and provide services in the patient’s home, primary care centers, and in both public and private nursing homes (Caroll et al., 2016).

In Lithuania, there were several examples of responsibilities being shifted in PHC teams, or other cases involving a differentiation of PHC activities. The current trend is to include new members on PHC teams (ie, secretaries) as well as to introduce new services at PHC institutions (ie, home-care, palliative care, informational offices), which replace activities traditionally seen as GP. District nurses changed their titles to community nurses (Jaruseviciene, et al., 2013).

In Portugal there were 18 community support teams in 2012. The composition of the team is three doctors, three nurses, a psychologist, a social worker, and an operational assistant. The acceptance criteria are: a resident in the Agrupamento de Centros de Saúde (ACES) area with a life-limiting disease and palliative care needs with curative or palliative disease treatment, a capable caregiver, the patient and family accept the care, and pain/symptom control. The team’s main activities are support for complex immobile patients in their homes, palliative medicine consultation, and PHC team counseling. They care for around 52 patients on an annual basis and give an additional 41 consultations.

Palliative care teams may include nurses, doctors, social workers, volunteers, chaplains, allied health practitioners, and a multitude of other therapists. Professional identities are clearly defined and team membership is secondary. Leadership is often hierarchical. Many practitioners function as the ‘wedges of a pie,’ each with their own clearly defined place in the overall care of the patient. The best models of service provision and leadership for palliative care have not yet been identified (Crawford and Sharonne, 2003).

Palliative care networks are available all over Belgium. They play an advisory role (but do not replace care). In Antwerp (a region with 750,000 inhabitants) the network consists of eight Full-time equivalent (FTE) nurses, 0.5 FTE GPs experienced in PC, 0.5 FTE psychologists, 2.5 FTE administrative staff, and one FTE coordinator. The main tasks performed by PC support teams are lending out supportive care mediation pumps, personalized advice in complex care situations, personalized pain reduction schemes, combinations of medicines, help at home for specific PC treatments such as the installation of supportive care infusion pumps, ascites tapping (paracentesis), palliative sedation, and handling principles of care surrounding euthanasia requests.

We can conclude that the size and composition of the team should be based on the needs assessment, clear task objectives, and careful job descriptions. Special home-care teams work in close collaboration with the PCT, the palliative care consult team, and palliative care units in nursing homes and residential homes. The palliative care consult team seeks to influence and improve the care for patients with advanced, incurable disease, and they are links between different levels of the health care system. Consult teams face specific challenges related to potential disagreements and conflicts with referring staff and the PCT. Late referrals mean that the team has to be particularly adaptable and innovative in order to ensure optimum patient care. The comprehensive care team is composed of various professionals: a social worker, a nurse, a chaplain, pharmacists, a psychologist, volunteers, and physicians. They address physical, emotional, and spiritual issues (Damen, et al., 2019). Most treatment and care at the end of life is delivered by multidisciplinary and multi-agency teams, working together to meet the needs of patients as they move between different health and social care settings and access different services (Bainbridge, et al., 2010; Luckett, et al., 2014).

### Barriers

PCTs are well placed to encourage early palliative care. Their long-term relationship with patients and their families may be a good basis for initiating timely arrangement of care. Yet, we found numerous barriers influencing PCTs in preparing care plans with patients (Groot, et al., 2005). There are many barriers (Claessen, et al., 2013; Almaawiy et al., 2014; Beernaert, et al., 2014; van Riet Paap, et al., 2014; Hawley, 2017; Shiraz et al., 2017):

- Lack of knowledge, skills, and experience relating to palliative care needs;
- Uncertainty and unpredictability of the illness trajectory/poor identification of patients requiring palliative care;
- GPs’ limited recognition and discussion on the end of life for organ-failure and old-age/dementia patients compared to cancer patients/episodic nature of non-cancer conditions can lead to a delayed recognition of the palliative transition;
- Suboptimal communication with patients;
- Suboptimal communication and collaboration with other health care professionals/lack of professional or specialist support;
- Limited time/high workload;
- Perceptions regarding the GP’s role in palliative care/role ambiguity (care/treatment versus coordination tasks);
- Perceptions regarding the definition of palliative care/limited public understanding of palliative care;
- Bureaucracy/financial systems not permitting reimbursements for palliative care.

### Facilitators

We identified many facilitators to improve the organization of palliative care. Similar facilitators regarding the organization of palliative care and useful tools exist in different countries (van Riet Paap, et al., 2014; Mistry, et al., 2015; Klok, et al., 2018, WHO, 2014):

- Education/training opportunities/extending the competency of the entire practice team/practice-oriented education;
- Education of the public/increasing political support and public advocacy campaigns;
- Tools for identification;
- Proactive, deliberate questioning about (non-acute) care needs;
- Continuity of care and multidisciplinary collaboration/networking;
- Acknowledging and mobilizing informal care;
- Using particular specializations;
- Team climate, professional guidance, network;
- Employment of practice assistants with additional qualifications;
- Multidisciplinary palliative care consultation teams that can offer GPs the opportunity to learn more, receive practical help, and also possibly receive emotional support in caring for specific patients at home;
- Financial arrangement, regulations;
- Sometimes, the same topic appears to be a facilitator in one country or region and a barrier in another.

### Palliative care education in PHC

Medical education in palliative care is essential to prepare future clinicians, nursing care providers, health care assistants, health care workers, home health aides, or home support workers. These providers care for increasingly complex patients who are dying at home and in residential care. Unfortunately, future clinicians and nurses continue to report palliative care as the area in which they experience distress and feel unprepared. Medical staff still do not receive adequate education in palliative care at the undergraduate or graduate level (Gibbins et al., 2010; Gamondi, et al., 2013; Head, et al., 2016; Pesut and Greig, 2017; Walker et al., 2017; Zelko et al., 2017).

In Slovenia, medical students have lectures about palliative care included in the final year of medical school, but the majority of palliative care training is included in graduate medical education. Capacity building programs such as ‘Step by step’, an organization of the Slovenian Palliative Medicine Society, offer additional training. Attendance at conferences on the topic are also believed to offer opportunities to bridge the gap in knowledge of palliative care.

EAPC guidelines published in 2007 recommend that teaching time for palliative care should prioritize pain and symptom management (55% of teaching time), psychosocial and spiritual aspects (20%), and communication skills (10%). Basic concepts, ethical and legal issues, and principles of teamwork and self-reflection are more minor topics, each receiving 5% of teaching time (Gamondi et al., 2013). There has been an increase in curricular offerings of palliative care education in undergraduate medical schools, and there appears to be greater consistency in the teaching content being delivered as part of palliative care curricula. Findings when compared to the EAPC recommendations suggest that, whereas concepts of pain management are being well addressed, current undergraduate curricula may not be adequately exploring issues of broader symptom control in palliative care and the psychosocial and spiritual aspects of care. Problems in education at the undergraduate level are lack of consistency in what undergraduates are taught about palliative care: teaching tends to be fragmented, ad hoc, and lacking in coordination, and that teaching tends to focus on the acquisition of knowledge and skills rather than attitudes.

Many graduate training programs consist of lectures and seminars in various aspects of palliative care, with little or no opportunity to gain clinical experience. An assessment of most of these programs is limited to the clinical expertise acquired during the course. To the best of our knowledge, no formal assessment of the impact of an educational program in palliative care in terms of emotional and communication coping abilities or in terms of attitude formation has been described. In general, we found that a comprehensive graduate training program in palliative care, integrated into a residency training program, is an effective way of teaching end-of-life care. Not only did the residents express a general feeling that their clinical skills and knowledge improved, but also a general positive trend in attitude was observed (Gibbins et al., 2014; Walker et al., 2017; Head, et al., 2016).

Training in a specialized palliative care context would increase capacity in essential palliative care skills in all European countries. Filling the gaps in the understanding of the pragmatic skills would prepare PCTs to integrate palliative care into their practices. Topics such as types of on-call systems and how to access home-care nurse practitioners should be included as well.

### Research on palliative care in PHC

Several challenges are known in both palliative care research and primary care research. The key challenges for palliative care research are mainly related to the functional decline and suffering experienced by palliative care patients, leaving little room for performing extra tasks such as participating in research. There is also the already-high care burden for family members and the gatekeeping function of health care professionals seeking to protect patients and their families from shouldering the respondent burden (Farquhar, et al., 2013; Leysen, et al., 2015; den Herder-van der Eerden et al., 2018).

The UK recognized palliative medicine as a specialty in 1987. There is national direction from the General Medical Council to implement palliative care training even at the general practitioner level. Learning outcomes and strategies are specified. Gibbins et al. surveyed the coordinators of palliative care teaching in 14 UK medical schools and found that the incorporation of palliative care into undergraduate medical education involved a complex process of individual, institutional, clinical, patient, and curricular factors. But still little is known about how palliative care training is delivered across UK medical schools.

So far, research has been conducted in specific patient groups with cancer, heart failure, COPD, dementia, frailty, end-stage liver disease, end-stage renal disease, and tuberculosis. There have been studies about symptom management (ie, pain treatment and opioid prescription, other symptoms, depression, and anxiety). Communication regarding end-of-life care and the development and use of tools for identifying patients in need of palliative care and determining and improving palliative care quality and self-management of symptoms was explored. Organizational aspects were discussed by exploring advanced care planning, continuity of care, out-of-hours care, multidisciplinary palliative care, palliative care in care homes/nursing homes, hospice care, and hospitalization. This research was mostly conducted in the United States, Canada, Australia, the UK, the Netherlands, Belgium, Spain (mostly Catalonia), and Germany.

### Call for action for policy and practice on local, national, and European level

The EFPC urges its members and partner organizations to become informed about the importance, value, and the movement for palliative care in primary care setting. This review demonstrates the clear benefits of home palliative care in helping patients and their families and care-givers in the process of dying at home with a reduced symptom burden and without a negative impact on caregiver grief.

The consensus-building process in the production of this position paper bears several challenges due to the considerable heterogeneity of service provision in different countries. This leads to the conclusion that common statements will reduce heterogeneity, but will also still encompass the various regional, geographic, and cultural focuses regarding the provision of palliative care. The consensus-building process has to take into account different European norms on cultural aspects of care provision in different countries and regions, which were also confirmed by the white paper on standards and norms for hospices (Gamondi, et al., 2013).

Good interprofessional collaboration between specialized palliative care teams and primary health teams (PHTs) often leads to workplace learning, in which both parties can absorb each other’s expertise and experience. New home palliative care interventions must respond to the challenges ahead, posed by rapidly aging populations with increased medical complexity and a growing need for home palliative care; these are international challenges (Gomes et al., 2012; 2013).

Until recently, little attention has been given to the development of a public health approach to palliative care at the end of life. The conclusions of series of workshops of the European forum for Primary care are:

- Palliative care is a humanitarian need.
- Effective palliative care services should be integrated into the existing health system, especially community and home-based care.
- PCTs should give terminally ill individuals and their families a greater sense of control and enable them to make informed decisions about their care.
- Caring for caregivers is an essential area of palliative care in primary care.
- Non-specialist palliative care needs should be considered by the staff delivering ongoing care, with initial guidance and support from specialists in hospitals and specialized palliative care teams.
- Interdisciplinary care that focuses on effective communication, individualized care plans, and care coordination should be implemented.
- The definition of essential requirements for primary care interventions throughout the palliative care pathway should be accepted by the profession at the international level.
- Communication between various professionals and sectors involved in palliative care, including via IT investment and integration of education, should be improved.
